# Responder analysis for neuropathic impairment and quality-of-life assessment in patients with hereditary transthyretin amyloidosis with polyneuropathy in the NEURO-TTR study

**DOI:** 10.1007/s00415-021-10635-1

**Published:** 2021-06-14

**Authors:** Aaron Yarlas, Andrew Lovley, Duncan Brown, Mark Kosinski, Montserrat Vera-Llonch

**Affiliations:** 1 QualityMetric, 1301 Atwood Avenue, Suite 216E, Johnston, RI 02919 USA; 2 Akcea Therapeutics, a Subsidiary of Ionis Pharmaceuticals, Boston, MA USA

**Keywords:** Neuropathy, Hereditary transthyretin amyloidosis with polyneuropathy, Clinically meaningful change, Responder definition, Responder analysis

## Abstract

**Objective:**

Hereditary transthyretin amyloidosis with polyneuropathy (ATTRv-PN) is a rare disease characterized by rapid neuropathic progression. In pivotal studies of gene-silencing treatments, the modified Neuropathy Impairment Score + 7 tests (mNIS + 7) and Norfolk-Quality of Life (QOL)-Diabetic Neuropathy (DN) questionnaire assessed treatment impact on neuropathic progression. Establishing responder definition (RD) thresholds for these measures would enable evaluation of clinically meaningful treatment benefit.

**Methods:**

mNIS + 7 and Norfolk-QOL-DN were administered at baseline and week 65 to 165 adults with ATTRv-PN receiving inotersen (*n* = 106) or placebo (*n* = 59) in the NEURO-TTR study. Anchor-based approaches for estimating RD thresholds were used for Norfolk QOL-DN, while distribution-based approaches were used for both measures. Responders were patients with a score change < RD, indicating improvement or stabilization (i.e., no clinically meaningful progression). Odds ratios (ORs) and Fisher’s exact tests compared proportions of responders by treatment.

**Results:**

The mean RD estimates were 12.2 points and 8.8 points for mNIS + 7 and Norfolk QOL-DN, respectively. The proportions of patients whose change in score indicated improvement or stabilization were statistically significantly larger for inotersen than placebo for all estimated RD thresholds for mNIS + 7 (64–86% responders for inotersen vs. 27–46% for placebo, ORs = 3.8–7.2, *p*s < 0.001) and Norfolk QOL-DN (66–81% vs. 35–56%, ORs = 2.4–3.6, *p*s < 0.05).

**Discussion:**

Establishing RD thresholds for these instruments enables evaluation of clinically relevant and individual-level treatment benefit on neuropathic progression. Across RDs estimated using multiple methods, a higher proportion of patients receiving inotersen than placebo showed improved or stabilized neuropathic progression at week 65.

**Trial registration:**

ClinicalTrials.gov Identifier: NCT01737398; Date of registration: November 29, 2012.

## Introduction

Hereditary transthyretin amyloidosis (ATTRv) is a rare, systemic, progressive, potentially fatal disease caused by mutations in the gene encoding the transthyretin (TTR) protein [1, 2]. More than 140 reported TTR gene mutations [3] promote misfolding of TTR proteins, which then aggregate and deposit as insoluble amyloid deposits in tissues, resulting in damage to the nervous system and multiple organs [4]. Patients with ATTRv commonly experience injury to the peripheral and autonomic nerve fibers, which can result in a length-dependent sensorimotor peripheral neuropathy, manifesting as pain, numbness, and weakness in the limbs and extremities, with a progressive impairment in ambulation [5, 6]. Patients with ATTRv associated with polyneuropathy (ATTRv-PN) experience diminished health-related quality of life (HRQOL) [7–9], which continues to decline as the disease progresses [10, 11].

Pivotal trials for two recently Food and Drug Administration (FDA)-approved gene-silencing treatments for ATTRv-PN—patisiran, an RNA interference therapy, and inotersen, an antisense oligonucleotide therapy—both included mean change in the modified Neuropathy Impairment Score + 7 test (mNIS + 7), a clinician-administered assessment of neuropathic impairment, as a primary efficacy endpoint, and mean change in total score of the Norfolk Quality of Life-Diabetic Neuropathy (Norfolk QOL-DN), a patient-reported questionnaire capturing neuropathy-related QOL, as a co-primary or key secondary efficacy endpoint [12, 13]. In the pivotal NEURO-TTR study that evaluated the safety and efficacy of 65 weeks of treatment with inotersen, mixed-effects models for repeated measures (MMRM) found statistically significant treatment benefits for inotersen, relative to placebo, on each of these measures, with placebo-corrected differences in mean changes for mNIS + 7 (− 19.7 points; 95% confidence interval [CI] − 26.4 to − 13.0; *p* < 0.001) and Norfolk QOL-DN total score (− 11.7 points; 95% CI − 18.3 to − 5.1; *p* < 0.001) [12].

No responder definition (RD) thresholds have been reported for the mNIS + 7 or Norfolk QOL-DN for patients with health conditions manifesting in neuropathy, including ATTRv-PN, which would enable﻿ evaluation of whether treatment benefit is clinically meaningful [14, 15]. A clinically meaningful treatment benefit can be observed when a patient’s change in a measured outcome exceeds some threshold that indicates a significant clinical improvement. This threshold—alternatively referred to as the RD, meaningful within-person change (MWC), or minimal clinically important difference (MCID)—has been defined as “the smallest difference in score in the domain of interest which patients perceive as beneficial and which would mandate, in the absence of troublesome side effects and excessive cost, a change in the patient's management” [16], or, more simply, “the smallest difference in a score that is considered to be worthwhile or important” [17].

An objective of the current analysis was to estimate RD thresholds for mNIS + 7 and Norfolk QOL-DN total scores in a sample of patients with ATTRv-PN in the NEURO-TTR study. Estimated RD thresholds were then used to compare the proportion of responders, classified as whether or not a patient’s change in score from baseline to the week 65 visit exceeded the estimated RD threshold, between patients receiving inotersen or placebo. Given that patients with ATTRv-PN experience rapidly progressing neuropathy [18], and that gene-silencing treatments can limit production of new TTR amyloid but not remove existing amyloid, the goal of these treatments is to slow or stop further neuropathic progression [19]. As a result, in this current analysis, a clinical responder was defined as a patient whose neuropathy did not meaningfully worsen over the 65-week treatment period, and as such, RD thresholds were estimated for increases in scores, reflecting increased impairment.

## Methods

### Study design and treatment

The NEURO-TTR study (ClinicalTrials.gov Identifier: NCT01737398) was a phase III, multinational, randomized, double-blinded, placebo-controlled study of inotersen in patients with ATTRv-PN. Patients were randomized in a 2:1 ratio to receive 65 weeks of treatment with either 300 mg subcutaneous inotersen (administered on 3 alternating days during the first week and once per week for the following 64 weeks) or placebo.

Key inclusion criteria included being at least 18 years old, classified in stage 1 (ambulatory without assistance) or stage 2 (ambulatory with assistance) neurologic disease severity [20], having a neuropathy impairment score (NIS) between 10 and 130 (inclusive) at screening, the presence of a TTR variant by genotyping, and evidence of amyloid deposits from biopsy. Exclusion criteria included previous or anticipated liver transplantation within 1 year of screening, a Karnofsky performance status ≤ 50, a New York Heart Association (NYHA) functional classification ≥ 3, or the presence of either type 1 or 2 diabetes, human immunodeficiency virus (HIV), hepatitis B, or hepatitis C.

Randomized allocation to treatment arm was stratified by presence/absence of previous treatment with tafamidis and/or diflunisal, TTR genetic mutation type (V30M or non-V30M), and neurologic disease severity stage (stage 1 or stage 2).

Co-primary endpoints of the study were changes from baseline to week 65 in mean mNIS + 7 and Norfolk QOL–DN total scores.

### Ethical standards

The NEURO-TTR study protocol was approved by the relevant institutional review boards or local ethics committees and regulatory authorities. The study was conducted in accordance with Good Clinical Practice guidelines of the International Conference on Harmonization and the principles of the Declaration of Helsinki. All patients provided written informed consent to participate in the study.

### Target measures

#### Modified neuropathy impairment score + 7 (mNIS + 7)

Neuropathic impairment was measured using the mNIS + 7 score [21, 22], a clinician-reported measure. The mNIS + 7 is calculated as the sum of two composite scores: the NIS composite score (37 items, score range: 0–244 points) and the modified + 7 composite score (26 items, score range: − 22.32 to 102.32 points). The NIS composite score is based on four domains: cranial nerves, motor strength/muscle weakness, reflexes, and sensation. The modified + 7 composite score is also based on four domains: autonomic nerve assessment, peripheral nerve assessment, sensory nerve assessment for touch-pressure, and sensory nerve assessment for heat-pain. The mNIS + 7 score ranges from − 22.32 to 346.32 points, with higher scores indicating greater neuropathic impairment. The normal deviates from heart rate, deep breathing, and nerve conduction were used in the calculation of mNIS + 7 for the current analysis. A more comprehensive description of the mNIS + 7 used in the NEURO-TTR study has been published elsewhere [21].

At both baseline and week 65 visits, the mNIS + 7 assessment was performed twice for each patient. Within each visit, the two assessments were conducted by the same clinician and occurred on separate days, with every effort made to conduct both assessments < 7 days apart. From the observed data, the median interval between assessments was 2.0 days (mean = 3.1 days; standard deviation [SD] = 3.1 days), ranging from 1 to 25 days. The average score across the two assessments was used for analysis; if only one assessment had been done, the single assessment was used in place of the average.

#### Norfolk quality-of-life-diabetic neuropathy (QOL-DN)

Neuropathy-related HRQOL was measured using the Norfolk QOL-DN questionnaire [23], a 35-item patient-reported outcome (PRO) measure that has been validated for use in patients with ATTRv-PN [24]. Items on the Norfolk QOL-DN allow for calculating a total score (ranging from − 4 to 136), based on scores for five domains capturing symptoms and impacts of neuropathic health: activities of daily living, autonomic neuropathy, large fiber neuropathy/physical functioning, small fiber neuropathy, and neuropathic symptoms. Higher scores indicate worse HRQOL.

For both the baseline and week 65 visits, the Norfolk QOL-DN was administered on the same day as the first assessment of the mNIS + 7. At all visits, the Norfolk QOL-DN was administered prior to administration of any other measures.

### Statistical analysis

All analyses described here were post hoc﻿ and exploratory. Analyses were based on the Full Analysis Set (FAS) of the NEURO-TTR study, which included all randomized patients who received at least one injection of the study drug (inotersen or placebo) and who had a baseline and at least one post-baseline efficacy assessment for the mNIS + 7 or Norfolk QOL-DN total score.

#### Estimation of responder definitions (RDs)

RD thresholds were estimated for the Norfolk QOL-DN using both anchor-based and distribution-based methods, and were estimated for the mNIS + 7 using distribution-based methods only, as there were no appropriate anchor measures available.

##### Anchor-based approaches

Anchor-based approaches are used to estimate RD thresholds based on the correspondence between change in the target measure (Norfolk QOL-DN) and changes in independent criterion measures, for which there are clearly defined indicators for interpreting change in a patient’s clinical health. Appropriate anchor measures assess similar constructs as those captured by the target measure, and changes in the anchor should have at least a moderate statistical association with the target: a correlation ≥ |0.30| between changes in the target measure and any anchor measure is recommended [25].

No appropriate anchors were identified for the mNIS + 7, as no other clinician-rated assessments of neuropathic impairment included in the NEURO-TTR study had well-established definitions indicating clinically meaningful improvement.

Two measures were identified as appropriate anchors to estimate RD thresholds for the Norfolk QOL-DN, each from the SF-36v2® Health Survey (SF-36v2), a 36-item PRO measure of generic HRQOL [26]. The first anchor measure was the general health (GH) item, asking respondents to characterize their health as “excellent,” “very good,” “good,” “fair,” or “poor.” A decrease of one step (e.g., from “very good” to “good,” or from “fair” to “poor”) from baseline to week 65 was interpreted as clinically meaningful change for this anchor. The second anchor measure was the SF-36v2 physical component summary (PCS) score, which captures global physical health. PCS score is calculated based on weighted sums of the eight SF-36v2 domains, with the domains weighted most heavily including physical function, role limitations due to physical health problems (role-physical), and bodily pain. PCS scores are expressed as *T*-scores using norm-based methods, standardized to a mean of 50 and a standard deviation of 10 in the general population. Higher SF-36v2 scores reflect better HRQOL. A change of 5 points, equivalent to one-half SD, is widely used as the RD threshold for PCS scores.

Spearman correlations between the changes in the GH item and PCS with changes in Norfolk QOL-DN from baseline to week 65 in the NEURO-TTR study were − 0.39 and − 0.54, respectively, supporting the use of these as anchor measures for estimating RD threshold for the Norfolk QOL-DN.

Two methods were used to estimate RD thresholds for the Norfolk QOL-DN from anchors. First, linear regression models were conducted for each anchor, with change in Norfolk QOL-DN as the outcome and change in the anchor measure as the predictor. The β-coefficient from each model represents the change in Norfolk QOL-DN corresponding to a one level/point change in the anchor measure. The β-coefficient was then multiplied by the anchor’s RD threshold (– 1 for the GH item, and – 5 for PCS) to estimate the change in Norfolk QOL-DN corresponding to a meaningful change in the anchor. Second, for each anchor measure, receiver-operating characteristic (ROC) curves were used to identify the optimal cut-off point on the Norfolk QOL-DN for classifying patients showing meaningful worsening or not based on the anchor measure (i.e., 1-level decrease vs. no change/increase for the GH item; ≥ 5-point decrease vs. < 5-point decrease for PCS). The optimal cut-off point was defined using the Index of Union (IU) method, which identifies the point for which the sensitivity and specificity values are simultaneously closest to the value of the area under the curve (AUC) [27].

##### Distribution-based approaches﻿

Distribution-based approaches for estimating RD thresholds are a function of the variability of scores, and in some cases, the reliability of scores, on a target measure. This approach uses statistical properties of scores on a target measure to estimate an RD threshold that exceeds a magnitude of change that could be accounted for by measurement error. In the current analysis, RD thresholds were estimated for mNIS + 7 and Norfolk QOL-DN scores using three distribution statistics: effect size (ES), standardized response mean (SRM), and standard error of measurement (SEM).

For ES, the mean change is set to 0.5, which is the value that corresponds to a moderate- or medium-sized difference according to Cohen’s recommended interpretations [28]. This value is then multiplied by the baseline SD (SD_baseline_). Systematic reviews of studies across several health conditions reported that RD thresholds estimated using an ES of 0.5, or 0.5*SD_baseline_, closely mapped to estimates using anchor-based approaches [29, 30].

The SRM is similar to the ES in that it characterizes standardized mean change, with the difference being that it is calculated based on variability of change in the measure over time. As with ES, SRM for mean change is set to 0.5. This value is then multiplied by the SD for changes in scores between these two visits (SD_change_).

The SEM captures the measurement error of a score by taking into account the variability of scores on the measure, as well as the measure’s reliability. Wyrwich and colleagues reported that one SEM of a measure had a magnitude similar to RD thresholds estimated using anchor-based approaches [31, 32]. The SEM is calculated by multiplying SD_baseline_ by the square-root of one minus the measure’s reliability. Reliability for the mNIS + 7 was estimated using the intraclass correlation coefficient (ICC) between the two assessments conducted at the baseline visit. ICC was calculated using Shrout and Fleiss’ (2,1) model [33], a two-way random-effects model appropriate for capturing intra-rater reliability when a single rater performs two assessments of the same target, when assuming that scores from raters are generalizable to the population of raters [34]. Reliability for Norfolk QOL-DN was calculated as Cronbach’s α for internal consistency among items at the baseline assessment.

#### Responder analysis

For each estimated RD threshold, and for the mean across all estimated RD thresholds, responder analyses compared the proportion of patients showing clinical response on the mNIS + 7 and the Norfolk QOL-DN between inotersen and placebo treatment arms at the week 65 visit﻿. Responders were defined as patients whose change in score < RD threshold, that is, their change in score did not indicate clinical worsening.

To be consistent with the primary efficacy analysis of the mNIS + 7 and Norfolk QOL-DN in the NEURO-TTR study, no imputation of missing values was performed in the primary responder analysis. As such, the primary responder analysis used a complete-case analysis, in which only patients with non-missing scores at the week 65 visit were included. However, to examine the robustness of results from the primary analysis when accounting for patient discontinuation, a sensitivity responder analysis was conducted for which any patient with a missing score value at week 65 was classified as a non-responder, representing the most conservative estimate for study drop-outs.

The proportions of responders were compared between inotersen and placebo treatment arms for each score, by each estimated RD threshold, using odds ratios (OR) with 95% confidence intervals (CI), with statistical significance tested using Fisher’s exact tests (two-tailed α). Because these analyses were exploratory, no adjustments were made to the familywise Type 1 error rate for multiple comparisons.

#### Empirical cumulative distribution function curves

Empirical cumulative distribution function (eCDF) curves, which show the percentage of patients with score changes below each observed value of change in a score from baseline to week 65 visits, were plotted for mNIS + 7 and Norfolk QOL-DN total scores. Separate curves were plotted for each treatment arm, with distances between curves on the *y*-axis at each point of the *x*-axis indicative of differences in the percentage of patients meeting each threshold of change.

## Results

### Patient characteristics

Baseline characteristics of the analysis sample by treatment arm are presented in Table 1. No statistically significant treatment differences were observed for any reported characteristics.

**Table 1:** Baseline patient characteristics in the NEURO-TTR study full analysis set (165)

Characteristic	Inotersen (*n* = 106)	Placebo (*n* = 59)	*P* value^a^
Age, mean (SD)	59.6 (12.4)	59.4 (14.1)	0.937
Sex, *N* (%)			0.861
Female	31 (29.2)	18 (30.5)	
Male	75 (70.8)	41 (69.5)	
Mutation type, *N* (%)			0.626
V30M	54 (50.9)	33 (55.9)	
Non-V30M	52 (49.1)	26 (44.1)	
Neuropathy stage, *N* (%)			0.605
Stage 1	71 (67.0)	42 (71.2)	
Stage 2	35 (33.0)	17 (28.8)	
Duration of neuropathic symptoms in years, mean (SD)	5.4 (4.5)	5.4 (4.4)	0.947
mNIS + 7 score, mean (SD)	79.4 (37.5)	74.1 (39.0)	0.399
Norfolk QOL-DN, mean (SD)	48.6 (28.2)	48.6 (27.0)	0.994

^a^*p* values (two-tailed) are based on independent-samples *t* tests for continuous variables and Fisher’s exact tests for categorical variables

*DN* diabetic neuropathy, *mNIS + 7* modified Neuropathy Impairment Score + 7, *QOL* quality of life, *SD* standard deviation

### Estimation of responder definition thresholds

RD thresholds estimated using anchor-based approaches for the Norfolk QOL-DN, and using distribution statistics for the mNIS + 7 and Norfolk QOL-DN, are reported in Table 2, along with score properties (i.e., SD_baseline_, SD_change_ from baseline to week 65, and reliability coefficients) used to calculate distribution-based statistics. The variability in RD thresholds across the different estimation statistics was fairly large for mNIS + 7, with a range of 12.1 points (6.9–19.0) and a coefficient of variation (CV) of 51%, but somewhat smaller for Norfolk QOL-DN, with a range of 7.5 points (6.4–13.8) and a CV of 29%. For distribution-based statistics, RD threshold estimates were largest for ES and smallest for SEM for both the mNIS + 7 and Norfolk QOL-DN. Anchor-based estimates for Norfolk QOL-DN yielded identical values across both anchors for each of the two methods: 7.2 points from the linear regression method, and 8.5 points from the ROC curve optimal cut-off point method. Mean values across all RD threshold estimates were 12.2 for mNIS + 7 and 8.8 for Norfolk QOL-DN.

**Table 2:** Responder definition estimates for mNIS + 7 and Norfolk QOL-DN Scores

	mNIS + 7	Norfolk QOL-DN
Statistics to calculate distribution-based RD thresholds		
Standard deviation at baseline	38.0	27.7
Standard deviation for change	21.5	20.4
Reliability coefficient	0.97	0.95﻿
Distribution-based estimates of RD thresholds		
Effect size	19.0	13.8
Standardized response mean	10.8	10.2
Standard error of measurement	6.9	6.4
Anchor-based estimates of RD thresholds		
SF-36v2 General Health item		
Linear regression	–	7.2
ROC curve optimal cut-off point^a^	–	8.5
SF-36v2 Physical Component Summary (PCS)		
Linear regression	–	7.2
ROC curve optimal cut-off point^a^	–	8.5
Summary for estimates of RD thresholds		
Mean	12.2	8.8
Standard deviation	6.2	2.5
Coefficient of variation	51%	29%

^a^Optimal cut-off points from ROC curves were selected using the Index of Union approach

*DN* diabetic neuropathy, *mNIS + 7* modified Neuropathy Impairment Score + 7, *QOL* quality of life, *RD* responder definition, *ROC* receiver-operating characteristic

### Responder analysis

Results from the primary responder analysis are presented in Table 3. For each estimated RD threshold for mNIS + 7, there was a statistically significantly larger proportion of responders in the inotersen arm (range 64–86%) than in the placebo arm (27–46%), ORs ranging from 3.8 to 7.2, all *p* < 0.001. A treatment benefit of inotersen was also observed for all estimated RD thresholds for the Norfolk QOL-DN total score, with a range of 66–81% responders for inotersen compared to 35–56% for placebo, ORs ranging from 2.4 to 3.6, all *p* < 0.05. Larger proportions of responders in the inotersen arm than in the placebo arm were observed at the mean RD value for both measures: the proportion of responders was twice as large for inotersen than placebo at the RD threshold of 12.2 points for mNIS + 7 (74% vs. 37%, OR = 5.1, *p* < 0.001) and 1.5 times as large at 8.8 points for Norfolk QOL-DN (67% vs. 46%, OR = 2.4, *p* = 0.020).

**Table 3:** Primary responder complete-case analysis for mNIS + 7 and Norfolk QOL-DN (*N* = 138)

Measure	RD type	RD value	Inotersen			Placebo			OR (95% CI)	*p*
			N	Responders (*n*)	Responders (%)	*N*	Responders (*n*)	Responders (%)		
mNIS + 7	Distribution									
	ES	19.0	86	74	86.0	52	24	46.2	7.2 (3.2, 16.3)	< 0.001
	SRM	10.8	86	59	68.6	52	19	36.5	3.8 (1.8, 7.8)	< 0.001
	SEM	6.9	86	55	64.0	52	14	26.9	4.8 (2.3, 10.2)	< 0.001
	﻿Mean RD	12.2	86	64	74.4	52	19	36.5	5.1 (2.4, 10.6)	< 0.001
Norfolk QOL-DN	Distribution									
	ES	13.8	85	69	81.2	52	29	55.8	3.4 (1.6, 7.4)	0.002
	SRM	10.2	85	60	70.6	52	26	50.0	2.4 (1.2, 4.9)	0.019
	SEM	6.4	85	56	65.9	52	18	34.6	3.6 (1.8, 7.5)	< 0.001
	Anchor									
	GH item									
	Regression	7.2	85	57	67.1	52	20	38.5	3.3 (1.6, 6.7)	0.001
	ROC cut-off	8.5	85	57	67.1	52	24	46.2	2.4 (1.2, 4.8)	0.020
	PCS									
	Regression	7.2	85	57	67.1	52	20	38.5	3.3 (1.6, 6.7)	0.001
	ROC cut-off	8.5	85	57	67.1	52	24	46.2	2.4 (1.2, 4.8)	0.020
	Mean RD	8.8	85	57	67.1	52	24	46.2	2.4 (1.2, 4.8)	0.020

*CI* confidence interval, *DN* diabetic neuropathy, *ES* effect size, *GH* SF-36v2 general health, *mNIS + 7* modified Neuropathy Impairment Score + 7, *PCS* SF-36v2 physical component summary, *OR* odds ratio, *QOL* quality of life, *RD* responder definition, *ROC* receiver-operating characteristic, *SEM* standard error of measurement, *SRM* standardized response mean

Results from the sensitivity responder analysis, presented in Table 4, were fairly similar to those from the primary analysis. As in the primary analysis, for each estimated RD threshold for mNIS + 7, and for the mean of RD thresholds (i.e., 12.2 points), there was a statistically significantly larger proportion of responders in the inotersen arm (range 52–70%) than in the placebo arm (24–41%), ORs ranging from 2.6–3.5, all *p* < 0.01. For the Norfolk QOL-DN, magnitudes of the proportion of responders were numerically larger at all RD thresholds in the inotersen arm (range 53–65%) than in the placebo arm (31–49%), with ORs ranging from 1.7 to 2.6, although these differences were not statistically significant for four of the seven estimated RD thresholds, nor for the mean RD threshold of 8.8 points (54% vs. 41%, OR = 1.7, *p* = 0.143).

**Table 4:** Sensitivity responder analysis for mNIS + 7 and Norfolk QOL-DN Scores (*N* = 165)

Measure	RD type	RDvalue	Inotersen			Placebo			OR (95% CI)	*p*
			*N*	Responders (*n*)	Responders (%)	*N*	Responders (*n*)	Responders (%)		
mNIS + 7	Distribution									
	ES	19.0	106	74	69.8	59	24	40.7	3.4 (1.7, 6.6)	< 0.001
	SRM	10.8	106	59	55.7	59	19	32.2	2.6 (1.4, 5.1)	0.005
	SEM	6.9	106	55	51.9	59	14	23.7	3.5 (1.7, 7.1)	< 0.001
	Mean RD	12.2	106	64	60.4	59	19	32.2	3.2 (1.6, 6.3)	< 0.001
Norfolk QOL-DN	Distribution									
	ES	13.8	106	69	65.1	59	29	49.2	1.9 (1.0, 3.7)	0.049
	SRM	10.2	106	60	56.6	59	26	44.1	1.7 (0.9, 3.1)	0.144
	SEM	6.4	106	56	52.8	59	18	30.5	2.6 (1.3, 5.0)	0.009
	Anchor									
	GH item									
	Regression	7.2	106	57	53.8	59	20	33.9	2.3 (1.2, 4.4)	0.015
	ROC cut-off	8.5	106	57	53.8	59	24	40.7	1.7 (0.9, 3.2)	0.143
	PCS									
	Regression	7.2	106	57	53.8	59	20	33.9	2.3 (1.2, 4.4)	0.015
	ROC cut-off	8.5	106	57	53.8	59	24	40.7	1.7 (0.9, 3.2)	0.143
	Mean RD	8.8	106	57	53.8	59	24	40.7	1.7 (0.9, 3.2)	0.143

*CI* confidence interval, *DN* diabetic neuropathy, *ES* effect size, *GH* SF-36v2 general health, *mNIS + 7* modified Neuropathy Impairment Score + 7, *PCS* SF-36v2 physical component summary, *OR* odds ratio, *QOL* quality of life, *RD* responder definition, *ROC* receiver-operating characteristic, *SEM* standard error of measurement, *SRM* standardized response mean

### eCDF curves

**Fig. 1:**
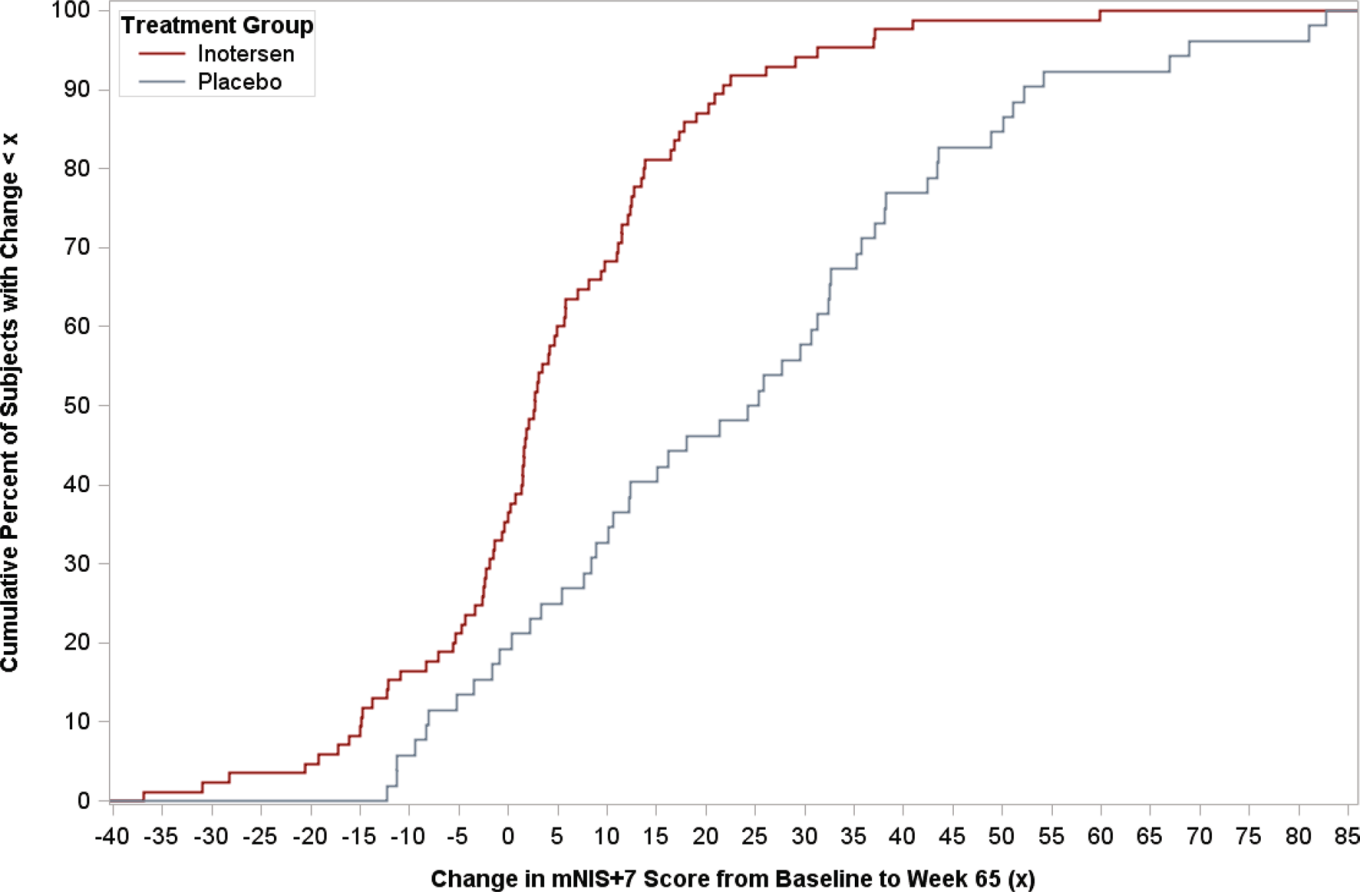
Empirical distribution function curve for change in mNIS + 7 score from baseline to week 65 by treatment arm

The eCDF curve for changes in mNIS + 7 from baseline to week 65 is presented in Fig. 1. Visual inspection of the curve shows that the percentages of patients with change scores below each value are larger for patients receiving inotersen than placebo across the entire range of change. The largest differences between the curves occur at changes between 5 and 30 points, where the percentages of patients receiving inotersen with change in scores below those points were approximately 30% higher than for patients receiving placebo. All estimated RD thresholds for mNIS + 7 were within this range.

**Fig. 2:**
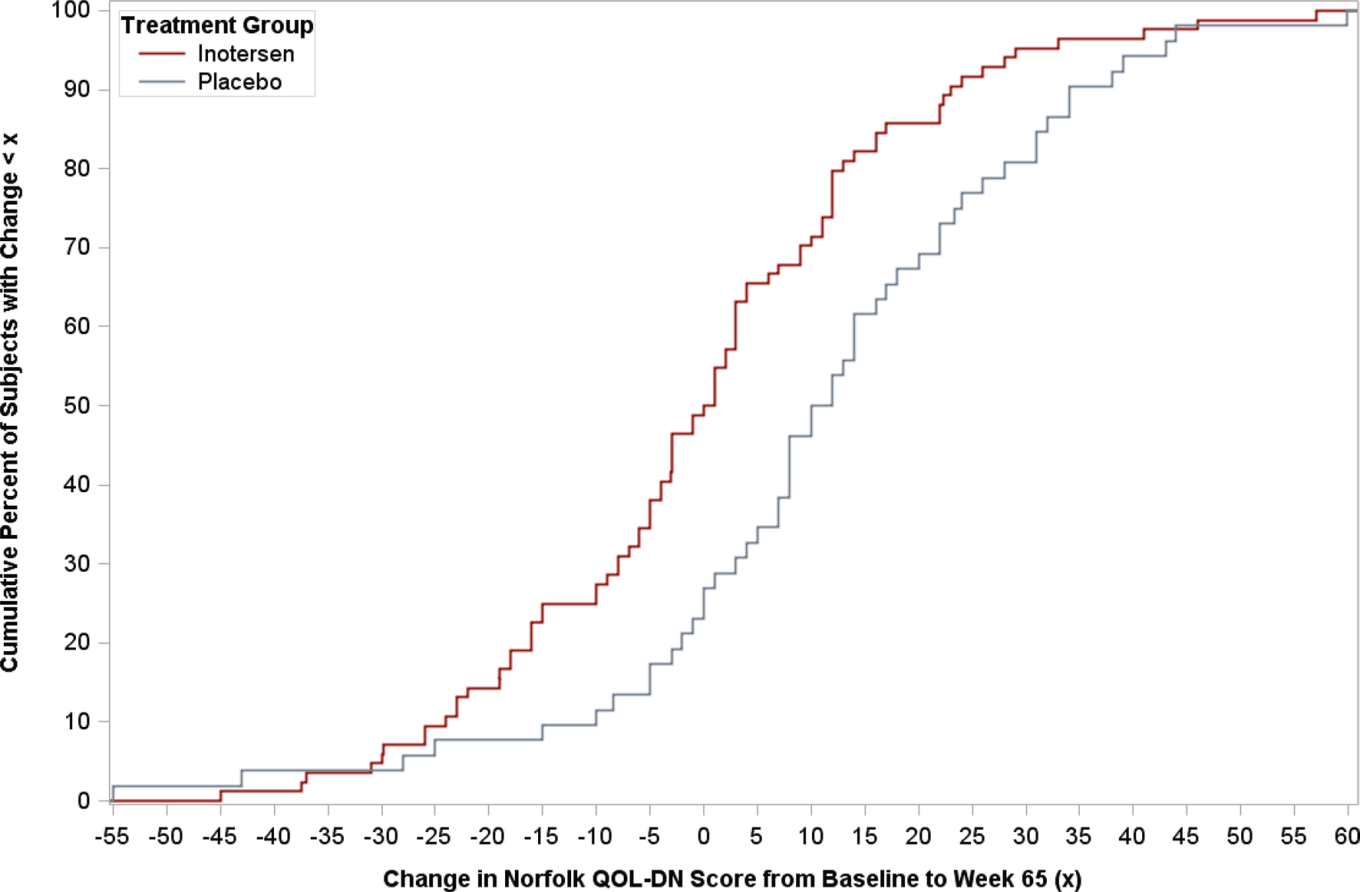
Empirical distribution function curve for change in Norfolk QOL-DN total score from baseline to week 65 by treatment arm

The eCDF curve for changes in Norfolk QOL-DN from baseline to week 65 is presented in Fig. 2. Visual inspection of the curve shows that, with the exception of the very tail end of values on the *x*-axis, the percentages of patients with change scores below each value are larger for inotersen than placebo across the range of change scores. The largest differences between the curves occur at changes between − 15 and 20 points, where the percentages of patients with change in scores below those points were approximately 20% higher for patients receiving inotersen as compared to placebo. All estimated RD thresholds for the Norfolk QOL-DN were within this range.

## Discussion

The mNIS + 7 and Norfolk QOL-DN have been and continue to be used as primary and/or secondary endpoints in pivotal trials examining therapeutic efficacy for slowing neuropathic progression in patients with ATTRv-PN. However, the clinical relevance of treatment benefit when using these measures has been limited by the lack of established thresholds indicating clinically meaningful change in neuropathic progression [14, 15].^1^ The current study is, to our knowledge, the first to estimate RD thresholds for either of these measures. Values for RD thresholds were estimated using both anchor-based and distribution-based approaches (or only the latter for mNIS + 7), and multiple methods or statistics within each of these approaches, allowing for triangulation across multiple estimates, which is generally considered best practice [25, 38, 39]. From the current analysis, based on the mean value across all estimates, the RD thresholds for indicating clinically meaningful worsening in neuropathy are 12.2 points for the mNIS + 7 and 8.8 points for the Norfolk QOL-DN.

Previous analyses showed statistically significant treatment benefit for inotersen on neuropathic impairment and neuropathy-specific QOL in patients with ATTRv-PN, with improvements in mean change of scores after 65 weeks of treatment, relative to placebo, of 19.7 points on mNIS + 7 and 11.7 points on Norfolk QOL-DN [12]. The magnitudes of placebo-corrected changes for inotersen are supportive of clinically meaningful benefit at the group level, with placebo-corrected changes for inotersen exceeding the means of RD thresholds estimated here.

The current analysis adds further evidence supporting this treatment benefit at the individual patient level, as indicated by a larger proportion of patients with stable or improved scores on the measures, and thus smaller proportions of patients showing clinically meaningful worsening, for each of these measures after 65 weeks of treatment with inotersen compared to placebo. Taken in sum, these findings indicate that the benefit of inotersen is not only statistically significant, but also clinically meaningful. Furthermore, these results indicate that treatment differences were not being driven by a minority of outliers showing extreme improvement or worsening, but rather reflect the experiences of the majority of study patients. Based on the mean of RD threshold estimates, approximately three-quarters of patients receiving inotersen (74%) showed stabilization of improvement on the mNIS + 7 compared to 37% of patients receiving placebo, while for Norfolk QOL-DN total scores, the proportions for the two groups were 67% vs. 46%, respectively. Thus, the majority of patients receiving inotersen experienced stabilization or improvement, in contrast with the majority of patients receiving placebo showing clinically meaningful worsening. Visual inspection of the eCDF plots for each of these measures shows that this benefit is consistent across a wide range of change in values on both measures.

Analyses in the current study were conducted using the entire sample of the NEURO-TTR study. However, as has been discussed elsewhere, it is quite possible that even within a single patient population, RD thresholds may vary as a function of patients’ disease severity, comorbidities, or other characteristics [40]. For example, it is possible that characteristics of the current sample, such as age of disease onset (early or late) and/or genetic mutation (V30M), predict treatment response, such that subgroups of patients with early onset V30M could experience a different magnitude of treatment response than patients with late-onset V30M. To examine such a case, we conducted a post hoc sensitivity responder analysis testing for differences in proportion of treatment responders, as defined by mean of RD threshold estimates (12.2 points for mNIS + 7, 8.8 points for Norfolk QOL-DN), between treatment arms for subgroups of complete-case patients with early onset V30M (*n* = 30) and late-onset V30M (*n* = 39). Patients with early onset V30M showed a substantial treatment benefit on both measures, with 94% and 81% responders for mNIS + 7 and Norfolk QOL-DN, respectively, for patients receiving inotersen compared to 29% and 14% for patients receiving placebo, with ORs of 37.5 and 26.0 and both *p*﻿ < 0.001. On the other hand, for patients with late-onset V30M, the proportion of responders did not statistically differ between treatment arms. For this group, the magnitude of treatment difference was sizable—65% responders for inotersen compared with 38% for placebo, OR = 3.1, Cohen’s *d*_probit_ = 0.71—but the small sample did not provide adequate statistical power to detect a difference (95% CI for OR 0.8, 11.8; *p* = 0.112). For the Norfolk QOL-DN, however, there were clearly no treatment differences in proportion of responders within this subgroup, mainly driven by a lack of progression observed in patients receiving placebo (with 75% responders, compared to 61% for inotersen, *p* = 0.495). It may be the case that inotersen provides more benefit for stabilizing neuropathic progression in patients with early onset V30M than late-onset V30M, possibly driven by a higher rate of progression in the former than the latter subgroup. It could also be the case that the RD thresholds estimated for the full sample are not applicable to patient subgroups, and that separate estimates should be conducted within each subgroup. While addressing this issue is beyond the scope of the manuscript, future research should examine whether the RD thresholds estimated here are appropriate to generalize to some or all key subgroups of patients with ATTRv-PN, and whether inotersen produces a clinical response within each of these subgroups.

In the current analysis, a meaningful change in PCS scores was defined as 5 points, or 0.5 SD, which has been widely used in other studies estimating anchor-based RD thresholds. However, based on results using distribution-based methods within a general population normative sample, the instrument’s developers proposed a change of 3.4 points as the RD threshold for PCS scores [26]. To examine the impact of using this value, rather than 5 points, to define meaningful change in PCS scores when estimating RD thresholds for the Norfolk QOL-DN, a sensitivity analysis was conducted using the 3.4 point value for PCS. Results were fairly similar: with the cut-off point of 3.4 for PCS, the RD threshold estimate using the linear regression method was smaller than with the cut-off point of 5 (4.9 vs. 7.2), with the same value found using the ROC curve optimal cut-off point method (8.5), resulting in a slightly smaller mean across all estimates (8.5 vs. 8.8). Responder analyses conducted using these estimates found results equivalent to those presented here (data not shown).

There are several limitations of the current analysis that warrant caution of the interpretability of the current findings. One limitation, specific to the mNIS + 7, was the use of only distribution-based estimates of RD thresholds, with no estimates from anchor-based approaches, which may be considered more informative than distribution-based approaches [41]. For example, the FDA PRO guidance ﻿document recommends determining meaningful within-patient change using anchor-based methods, with distribution-based methods as secondary or supportive [42]. Unfortunately, there were no appropriate anchors (e.g., physician global impression of severity or change, or a “gold-standard” clinical assessment) included in the NEURO-TTR study, precluding this approach. RD thresholds estimated from anchor-based approaches in other longitudinal studies of patients with ATTRv-PN would provide additional support for establishing an RD estimate for the mNIS + 7 in this patient population.

Another limitation of the current analysis is the large variability across RD thresholds estimated using different statistics for each of the measures, particularly for the mNIS + 7. For the mNIS + 7, the CV across estimates was 51%, with the magnitude of the largest estimated threshold approximately three times larger than the smallest, while for the Norfolk QOL-DN, the CV across estimates was 29%, with the magnitude of the largest estimated threshold approximately twice as large as the smallest. While some variability across estimates is to be expected, and while reporting a range of plausible RD thresholds derived from multiple methods is preferred to a single, possibly arbitrarily determined number [17, 38], the large variation among estimates observed here provides less confidence that these methods have identified the “true” threshold of meaningful change for each measure. At the same time, there was some convergence across estimates, particularly for the anchor-based estimates for the Norfolk QOL-DN, which yielded identical values across the two anchors for both methods (i.e., linear regression models and ROC curve optimal cut-off points). It should also be noted that treatment benefit was consistent across the entire range of estimated thresholds for both measures, which corresponds to findings on the eCDF curves of benefit across the range of changes on these measures. Still, estimation of RD thresholds for these measures within other samples of patients with ATTRv-PN would provide a greater level of precision, and thus a higher level of certainty, as to the value that best represents the “true” threshold of change.

An additional limitation of this study is that, as pointed out in previous research, the threshold representing a meaningful change on a measure, particularly for non-linear scales such as those examined here, may vary as a function of a patient’s baseline severity [17, 43]. For example, one study found that patients with more severe initial low back pain required greater change to be deemed clinically important than those with less severe initial pain [44]. This phenomenon could be related to floor or ceiling effects (a patient with lower initial severity has the opportunity to experience more disease progression than a patient with initial higher initial severity) or due to differing interpretations of differences in meaning across the continuum of the construct captured by the scale. As such, it cannot be assumed that the RD thresholds estimated here represent clinically meaningful differences on these scales for patients at all levels of disease severity and progression, and future research should examine this further.

In conclusion, the current study, using data from the NEURO-TTR study of patients with ATTRv-PN, yielded estimates for RD thresholds of mNIS + 7 (mean estimate: 12.2 points) and Norfolk QOL-DN (8.8 points). Furthermore, across all estimated RD thresholds for both measures, results showed a clear and consistent treatment benefit for inotersen in these patients, with significantly fewer of these patients showing clinically meaningful progression over a period of 65 weeks relative to patients receiving placebo. These results are supportive of previous findings demonstrating efficacy of inotersen for stabilizing neuropathic progression in this patient population.

## Data Availability

Data can be made available upon reasonable request.

## Footnotes

1 While Benson et al. [12] suggested that a two-point change in mNIS + 7 reflected a minimal clinically important difference, this value was in fact recommended as the RD threshold for the NIS or NIS-Lower Limbs (NIS-LL) assessments, [35–37] which have substantially smaller ranges of scores than does the mNIS + 7 (88 for NIS-LL, 244 for NIS, and 368.64 points for mNIS + 7).

## Code availability

Statistical code can be made available upon reasonable request.

## References

[1] Ando Y, Coelho T, Berk JL (2013). Guideline of transthyretin-related hereditary amyloidosis for clinicians. Orphanet J Rare Dis.

[2] Hawkins PN, Ando Y, Dispenzeri A (2015). Evolving landscape in the management of transthyretin amyloidosis. Ann Med.

[3] Rowczenio DM, Noor I, Gillmore JD (2014). Online registry for mutations in hereditary amyloidosis including nomenclature recommendations. Hum Mutat.

[4] Sekijima Y (2015). Transthyretin (ATTR) amyloidosis: clinical spectrum, molecular pathogenesis and disease-modifying treatments. J Neurol Neurosurg Psychiatry.

[5] Gertz MA (2017). Hereditary ATTR amyloidosis: burden of illness and diagnostic challenges. Am J Manag Care.

[6] Planté-Bordeneuve V, Said G (2011). Familial amyloid polyneuropathy. Lancet Neurol.

[7] Lovley A, Raymond K, Guthrie SD (2021). Patient-reported burden of hereditary transthyretin amyloidosis on functioning and well-being. J Patient-Rep Outcomes.

[8] Stewart M, Shaffer S, Murphy B (2018). Characterizing the high disease burden of transthyretin amyloidosis for patients and caregivers. Neurol Ther.

[9] Yarlas A, Gertz MA, Dasgupta NR (2019). Burden of hereditary transthyretin amyloidosis on quality of life. Muscle Nerve.

[10] Coelho T, Vinik A, Vinik EJ (2017). Clinical measures in transthyretin familial amyloid polyneuropathy. Muscle Nerve.

[11] Inês M, Coelho T, Conceição I (2020). Health-related quality of life in hereditary transthyretin amyloidosis polyneuropathy: a prospective, observational study. Orphanet J Rare Dis.

[12] Benson MD, Waddington-Cruz M, Berk JL (2018). Inotersen treatment for patients with hereditary transthyretin amyloidosis. N Engl J Med.

[13] Adams D, Gonzalez-Duarte A, O’Riordan WD (2018). Patisiran, an RNAi therapeutic, for hereditary transthyretin amyloidosis. N Engl J Med.

[14] Lasser K, Hoch JS, Mickle K et al. (2018) Inotersen and patisiran for hereditary transthyretin amyloidosis: effectiveness and value. Final evidence report. https://www.icer-review.org/wp-content/uploads/2018/02/ICER_Amyloidosis_Final_Evidence_Report_100418.pdf. Published 4 Oct 2018

[15] Mickle K, Lasser KE, Hoch JS (2019). The effectiveness and value of patisiran and inotersen for hereditary transthyretin amyloidosis. J Manag Care Spec Pharm.

[16] Jaeschke R, Singer J, Guyatt GH (1989). Measurement of health status. Ascertaining the minimal clinically important difference. Control Clin Trials.

[17] Hays RD, Woolley JM (2000). The concept of clinically meaningful difference in health-related quality-of-life research. How meaningful is it?. Pharmacoeconomics.

[18] Lin X, Yarlas A, Vera-Llonch M (2021). Rate of neuropathic progression in hereditary transthyretin amyloidosis with polyneuropathy and other peripheral neuropathies: a systematic review and meta-analysis. BMC Neurol.

[19] Luigetti M, Romano A, Di Paolantonio A (2020). Diagnosis and treatment of hereditary transthyretin amyloidosis (hATTR) polyneuropathy: current perspectives on improving patient Care. Ther Clin Risk Manag.

[20] Coutinho P, Martins da Silva A, Lopes Lima J, Glenner G, Costa P, de Freitas A (1980). Forty years of experience with type 1 amyloid neuropathy. Review of 483 cases. Amyloid and amyloidosis.

[21] Dyck PJ, Kincaid JC, Dyck P (2017). Assessing mNIS + 7 Ionis and international neurologists' proficiency in a familial amyloidotic polyneuropathy trial. Muscle Nerve.

[22] Suanprasert N, Berk JL, Benson MD (2014). Retrospective study of a TTR FAP cohort to modify NIS + 7 for therapeutic trials. J Neurol Sci.

[23] Vinik EJ, Hayes RP, Oglesby A (2005). The development and validation of the Norfolk QOL-DN, a new measure of patients' perception of the effects of diabetes and diabetic neuropathy. Diabetes Technol Ther.

[24] Vinik EJ, Vinik AI, Paulson JF (2014). Norfolk QOL-DN: validation of a patient reported outcome measure in transthyretin familial amyloid polyneuropathy. J Peripher Nerv Syst.

[25] Revicki D, Hays RD, Cella D (2008). Recommended methods for determining responsiveness and minimally important differences for patient-reported outcomes. J Clin Epidemiol.

[26] Maruish ME (2011). User's manual for the SF-36v2 health survey.

[27] Unal I (2017). Defining an optimal cut-point value in ROC analysis: an alternative approach. Comput Math Methods Med.

[28] Cohen J (1998). Statistical power analysis for the behavioral sciences.

[29] Norman GR, Sloan JA, Wyrwich KW (2003). Interpretation of changes in health-related quality of life: the remarkable universality of half a standard deviation. Med Care.

[30] Norman GR, Sloan JA, Wyrwich KW (2004). The truly remarkable universality of half a standard deviation: confirmation through another look. Expert Rev Pharmacoecon Outcomes Res.

[31] Wyrwich KW, Nienaber NA, Tierney WM (1999). Linking clinical relevance and statistical significance in evaluating intra-individual changes in health-related quality of life. Med Care.

[32] Wyrwich KW, Tierney WM, Wolinsky FD (1999). Further evidence supporting an SEM-based criterion for identifying meaningful intra-individual changes in health-related quality of life. J Clin Epidemiol.

[33] Shrout PE, Fleiss JL (1979). Intraclass correlations: uses in assessing rater reliability. Psychol Bull.

[34] Koo TK, Li MY (2016). A guideline of selecting and reporting intraclass correlation coefficients for reliability research. J Chiropr Med.

[35] Coelho T, Maia LF, da Silva M, Ana, (2012). Tafamidis for transthyretin familial amyloid polyneuropathy: a randomized, controlled trial. Neurology.

[36] Asbury AK, Porte D (1992). Proceedings of a consensus development conference on standardized measures in diabetic neuropathy. Neurology.

[37] Dyck PJ, Davies JL, Litchy WJ (1997). Longitudinal assessment of diabetic polyneuropathy using a composite score in the Rochester Diabetic Neuropathy Study cohort. Neurology.

[38] Guyatt GH, Osoba D, Wu AW (2002). Methods to explain the clinical significance of health status measures. Mayo Clin Proc.

[39] Yost KJ, Eton DT (2005). Combining distribution- and anchor-based approaches to determine minimally important differences: the FACIT experience. Eval Health Prof.

[40] Terwee CB, Roorda LD, Dekker J (2010). Mind the MIC: large variation among populations and methods. J Clin Epidemiol.

[41] Wyrwich KW, Norquist JM, Lenderking WR (2013). Methods for interpreting change over time in patient-reported outcome measures. Qual Life Res.

[42] U.S. Food and Drug Administration (2009) Guidance for industry, patient-reported outcome measures: use in medical product development to support labeling claims. Available from: https://www.fda.gov/media/77832/download. Accessed 3 Jan 2021 10.1186/1477-7525-4-79 PMC1629006 17034633

[43] Crosby RD, Kolotkin RL, Williams G (2003). Defining clinically meaningful change in health-related quality of life. J Clin Epidemiol.

[44] Stratford PW, Binkley JM, Riddle DL (1998). Sensitivity to change of the Roland–Morris Back Pain Questionnaire: part 1. Phys Ther.

